# Covalent dye attachment influences the dynamics and conformational properties of flexible peptides

**DOI:** 10.1371/journal.pone.0177139

**Published:** 2017-05-23

**Authors:** Manuel P. Luitz, Anders Barth, Alvaro H. Crevenna, Rainer Bomblies, Don C. Lamb, Martin Zacharias

**Affiliations:** 1 Department Physik, T38, Technische Universität München, 85748 Garching, Germany; 2 Department Chemie, Physikalische Chemie, Ludwig-Maximilians-Universität München, 81377 München, Germany; 3 Instituto de Tecnologia Química e Biológica António Xavier, Universidade Nova de Lisboa, 2780-157 Oeiras, Portugal; Friedrich-Alexander-Universitat Erlangen-Nurnberg, GERMANY

## Abstract

Fluorescence spectroscopy techniques like Förster resonance energy transfer (FRET) and fluorescence correlation spectroscopy (FCS) have become important tools for the *in vitro* and *in vivo* investigation of conformational dynamics in biomolecules. These methods rely on the distance-dependent quenching of the fluorescence signal of a donor fluorophore either by a fluorescent acceptor fluorophore (FRET) or a non-fluorescent quencher, as used in FCS with photoinduced electron transfer (PET). The attachment of fluorophores to the molecule of interest can potentially alter the molecular properties and may affect the relevant conformational states and dynamics especially of flexible biomolecules like intrinsically disordered proteins (IDP). Using the intrinsically disordered S-peptide as a model system, we investigate the impact of terminal fluorescence labeling on the molecular properties. We perform extensive molecular dynamics simulations on the labeled and unlabeled peptide and compare the results with *in vitro* PET-FCS measurements. Experimental and simulated timescales of end-to-end fluctuations were found in excellent agreement. Comparison between simulations with and without labels reveal that the *π*-stacking interaction between the fluorophore labels traps the conformation of S-peptide in a single dominant state, while the unlabeled peptide undergoes continuous conformational rearrangements. Furthermore, we find that the open to closed transition rate of S-peptide is decreased by at least one order of magnitude by the fluorophore attachment. Our approach combining experimental and *in silico* methods provides a benchmark for the simulations and reveals the significant effect that fluorescence labeling can have on the conformational dynamics of small biomolecules, at least for inherently flexible short peptides. The presented protocol is not only useful for comparing PET-FCS experiments with simulation results but provides a strategy to minimize the influence on molecular properties when chosing labeling positions for fluorescence experiments.

## Introduction

Structural changes of biomolecules are often closely related to their function. For proteins, usually large rearrangements of domains occur during the functional cycle. In the family of intrinsically disorderd proteins (IDP) certain regions undergo continuous structural changes. The transition from disorder to order is often part of the biological function mechanism of these proteins but may otherwise be an artifact of the genetic evolution [1]. Due to the lack of stable structure, they elude conventional structural biology approaches. Fluorescence spectroscopy techniques provide a useful toolbox to investigate the dynamics and extent of structural rearrangements of biomolecules *in vitro* and *in vivo* [2–6]. One of the most common approaches is the use of Förster resonance energy transfer (FRET) between two fluorophores attached to the molecule of interest [7]. The radiationless transfer of energy from the excited donor dye to the red-shifted acceptor dye depends on the relative orientation and distance between the fluorophores. Its high sensitivity in the range of 20–100 Å renders the effect interesting for experimental determination of distances on the molecular scale. Another example of radiation-less energy transfer is photoinduced electron transfer (PET) [2, 3, 8–11]. The excited-state energy may dissipate via electron transfer from the fluorophore to the quencher or vice versa, depending on the redox potentials of the excited state fluorophore and quencher. Relaxation to the ground state then occurs non-radiatively by charge recombination of the radical donor/acceptor ion pair. The timescale of the PET reaction resides in the range of femtoseconds to picoseconds [12, 13] which is significantly faster than the fluorescence lifetime of the fluorophores of typically a few nanoseconds [14, 15]. The efficiency of PET decays exponentially with distance on the length scale of a few Å, showing effectively an all-or-nothing quenching behaviour.

PET enables the *in vitro* time resolved detection of closed and open contacts between fluorophore and quencher in proteins and other biomolecules. Due to the on-off characteristics of PET, it is commonly combined with fluorescence correlation spectroscopy (FCS) [16] to study the timescale of the dynamic changes of the fluorescence signal. FCS is based on the analysis of the time correlation of the detected signal and is thus sensitive to all processes that effect the fluorescence signal. Most commonly, FCS is being used to study the diffusion properties of molecules [17], but it is also a powerful tool to study conformational dynamics when combined with FRET or PET [18, 19].

In biomolecules, the strongest fluorescence quenching of commonly used fluorophores for PET (e.g. Atto655, MR121 and Atto Oxa11) is observed for tryptophan in proteins and guanine in nucleic acids due to their oxidation potentials that allow them to act as electron donors [20, 21]. If tryptophan is not part of the protein sequence, PET experiments can be performed by engineering a Trp residue in the region of interest by either mutation or the adherence of an additional residue. On the other hand, Trp residues which might interfere in an undesirable way with the fluorophore need to be deleted from the protein. The labeling of proteins with fluorophores usually requires modification of the protein sequence. Typically, labeling is performed by reacting a maleimide derivative of the fluorescent dye with cysteine residues in the protein. To achieve specificity, this approach requires the removal of natural cysteine residues or the introduction of additional cysteine residues by mutation. Other labeling approaches target amino groups or rely on bioorthogonal labeling strategies based on the addition of unnatural amino acids [22]. After attaching the fluorophore to the molecule of interest, careful control measurements have to be performed to ensure that the photophysical properties are not altered by the local environment, and that no sticking interactions occur which would impair the rotational freedom of the fluorophore.

Although fluorescence techniques have been applied successfully on a variety of systems [5, 23, 24], usually little information is available to what extent the structural or dynamical properties of the studied system are modified by the attachment of the fluorescence labels. Molecular dynamics studies can help in the interpretation of experimental results and detection of potential artifacts introduced by the dye label [25–27]. As the fluorophores typically exceed the size of naturally occurring amino acids, it is expected that at least the local diffusivity is modified. Furthermore, many readily available fluorophores contain rigid ring systems which function as light absorbing centers (e.g. oxazine derivatives MR121, Atto655 and Atto Oxa11) and potentially facilitate hydrophobic or *π*-stacking interactions with other aromatic ring structures especially of the quencher. A significant influence of the fluorescent label on the local structure, the conformational dynamics and the overall functionality of the protein can thus usually not be excluded, and careful controls have to be performed to ensure the validity of the experimental results.

In the present study we conducted a comparative *in vitro* and *in silico* study on the 14 amino acid long truncated S-peptide, which historically served as a model system for intrinsically disordered peptides [28–32]. S-peptide is a fragment of RNAse-S formed after proteolytic cleavage of bovine Ribonuclease A by the subtilisin protease [33, 34]. While S-peptide is intrinsically disordered in solution, it adopts a stable helical fold upon association to S-protein forming the complex RNAse-S [32]. We N-terminally attached fluorophore Atto655 and added a tryptophan residue to the C-terminus serving as a fluorescence quencher [2]. For the labeled peptide, the fluorescence quenching autocorrelation was measured and analyzed with respect to the dynamic contribution, which is a measure for the end-to-end dynamics of the peptide chain. Additionally, we performed extensive, continuous molecular dynamics (MD) simulations on S-peptide with and without the fluorescence labels, to provide atomistic insight into the dynamics and sampled conformational regimes of the peptides.

Quantitative agreement of the experimental quenching autocorrelation of labeled S-peptide and *in silico* results was obtained. Comparison of the simulations reveals, that the dynamical and conformational regime of the flexible S-peptide was significantly altered by the attachment of Atto655 and Trp15. This study sheds light on systematic modifications of macromolecular properties introduced by fluorescence labeling and provides valuable insight for the design of future fluorescence spectroscopy experiments.

## Results

### MD simulations

**Fig 1:**
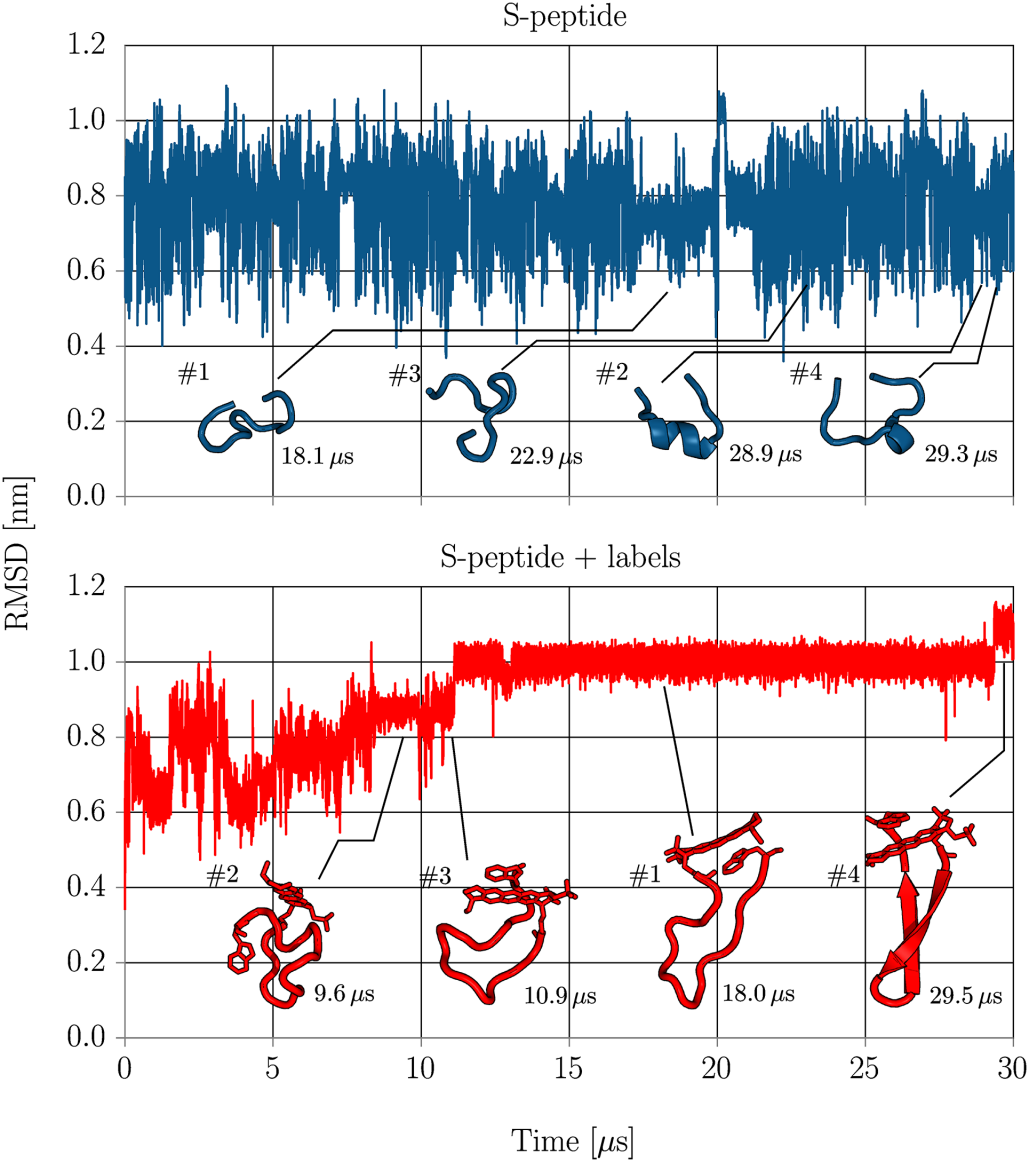
The RMSD of non-hydrogen (heavy) atoms of residues 1-14 with respect to the unfolded starting structure for simulations with (lower panel) and without (upper panel) labels. The mean structures of the respectively four largest clusters are shown and their cluster index is indicated (#). Additionally, the simulation time when the clusters mean structures were observed during the simulation is indicated at the bottom right of each cluster structure.

Extensive molecular dynamics simulations were performed for the labeled and unlabeled versions of the S-peptide over 30 μs for each trajectory. Both trajectories were started from extended peptide configurations. To give qualitative insight in the folding dynamics of intrinsically disordered S-peptide, the evolution of the RMSD with respect to the starting structure was calculated (Fig 1) and trajectories were visually inspected. To allow direct comparison between RMSD regimes of both systems, the RMSD was only calculated for residues 1 to 14 (without Atto655 and Trp15).

**Fig 2:**
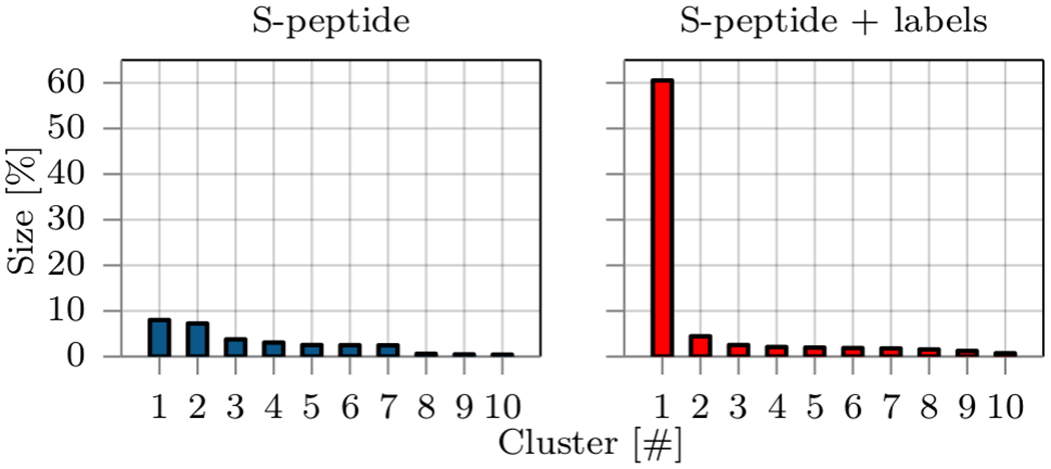
The population size of the ten largest clusters in percentage of lifetime compared to the whole trajectory from simulations without (left panel) and with (right panel) fluorescence labels. Clustering was based on the RMSD and the single linkage algorithm [35] with a 0.25 nm cutoff was used.

Unlabeled S-peptide rapidly fluctuated between conformational modes on the timescale of several nanoseconds as expected for a intrinsically disordered peptide. Fluctuation of the RMSD indicated no stable conformation surviving in the microseconds time regime throughout the whole simulation. Labeled S-peptide, however, showed a significantly reduced bandwidth of RMSD fluctuations with several plateaus in the RMSD evolution. Visual inspection confirmed metastable states surviving for several microseconds during the trajectory. Many configurations revealed close contacts between the two ring systems of terminal Atto655 and Trp15 indicating a strong stacking interaction that traps the system in a quenched state. After about 12 μs, the backbone locked into a stable *β*-sheet like configuration and remained in this state until finally folding to a *β*-sheet structure after 29 μs (see Figs 1 and 2 and conformational regime clusters #1 and #4).

### Sampled conformational regimes

Conformations from MD trajectories were clustered for both systems separately to quantify the impact of labeling on the relevant conformational regimes. Clustering along the RMSD was performed with the single-linkage algorithm using a RMSD cutoff of 0.25 nm and 10^5^ frames from each trajectory [35]. Clusters were sorted and numbered by their frequency of occurrence and the distribution of the ten biggest clusters is shown in Fig 2.

As expected from visual inspection of the trajectories, cluster sizes of unmodified S-peptide reflect the typical conformational behavior of an intrinsically disordered peptide. The decrease in probability with increasing cluster index is relatively moderate suggesting low free energy differences between neighboring clusters. A total of about 4700 clusters was found and the S-peptide adopted conformations belonging to the largest cluster only during 6% of the total simulation time. The mean structures of the first four clusters are depicted in Fig 1 and give insight into the variability of conformations.

With the attachment of Atto655 and Trp15 to the termini of S-peptide, the conformational behavior however changed significantly. The variability of clusters narrowed down to about 1000 different clusters, with many showing stacked Atto655/Trp15 configurations. The largest cluster, found between 12–29 μs, dominates the accessible conformational regimes with a probability of over 60% and indicates a shift from intrinsic disorder to a metastable folded peptide, reducing the conformational variability significantly. Three out of four mean structures of the largest clusters show strong stacking interaction between terminal labels (Fig 1). Interestingly, the conformation of the largest cluster #1 was never adopted by the trajectory of S-peptide without labels (according to the 0.25 nm threshold used for clustering). The closest structure had a distance of RMSD = 0.30 nm. As it is unlikely that this finding is attributed to insufficient sampling (see section conformational dynamics), it is a strong indicator that the attachment of the labels creates a deep minimum in the conformational landscape that is not present for the unlabeled peptide. Note, even within the first 12 μs, the sampled states of the S-peptide with terminal labels showed only little overlap with the most populated clusters of the S-peptide simulation without dyes. None of the sampled conformations approached the mean structure of the 10 most populated clusters of the S-peptide simulation without terminal dyes to less than the cluster RMSD cutoff of 0.25 nm (smallest deviation 0.28 nm).

### Conformational dynamics

To characterize the effect of the fluorescent labels on the conformational dynamics of the S-peptide, we compare the simulations with and without fluorescence labels by defining a two-state model (open/close) based on the end-to-end distance *d* of the peptide. The distance *d* was calculated between C-*β* atoms of residues Lys1 and Asp14 again for both systems with and without labels. We split the distance ensemble in two regimes, to characterize the switching dynamics between a structured and disordered regime of S-peptide. Distances *d* < 1.3 nm were assigned to a “close” regime while distances *d* > 2.5 nm were assigned to an “open” regime. By counting the number of transition (*N*_*T*_) from one regime to the other and dividing it by the total simulation time, a mean rate of opening and closing events of the peptide was calculated. For the S-peptide without labels, a open to closed transition rate of 50.1 μs^−1^ was found (*N*_*T*_ = 1503 in 30 μs), while refolding dynamics for labeled S-peptide were slowed by more then one order of magnitude to 6.6 μs^−1^ during the initial 12 μs (*N*_*T*_ = 80). Not a single open to close transition event was observed in the simulation interval 12–30 μs (*N*_*T*_ = 0) confirming the strong trapping of labeled S-peptide in this conformational regime.

### Circular dichroism spectra

**Fig 3:**
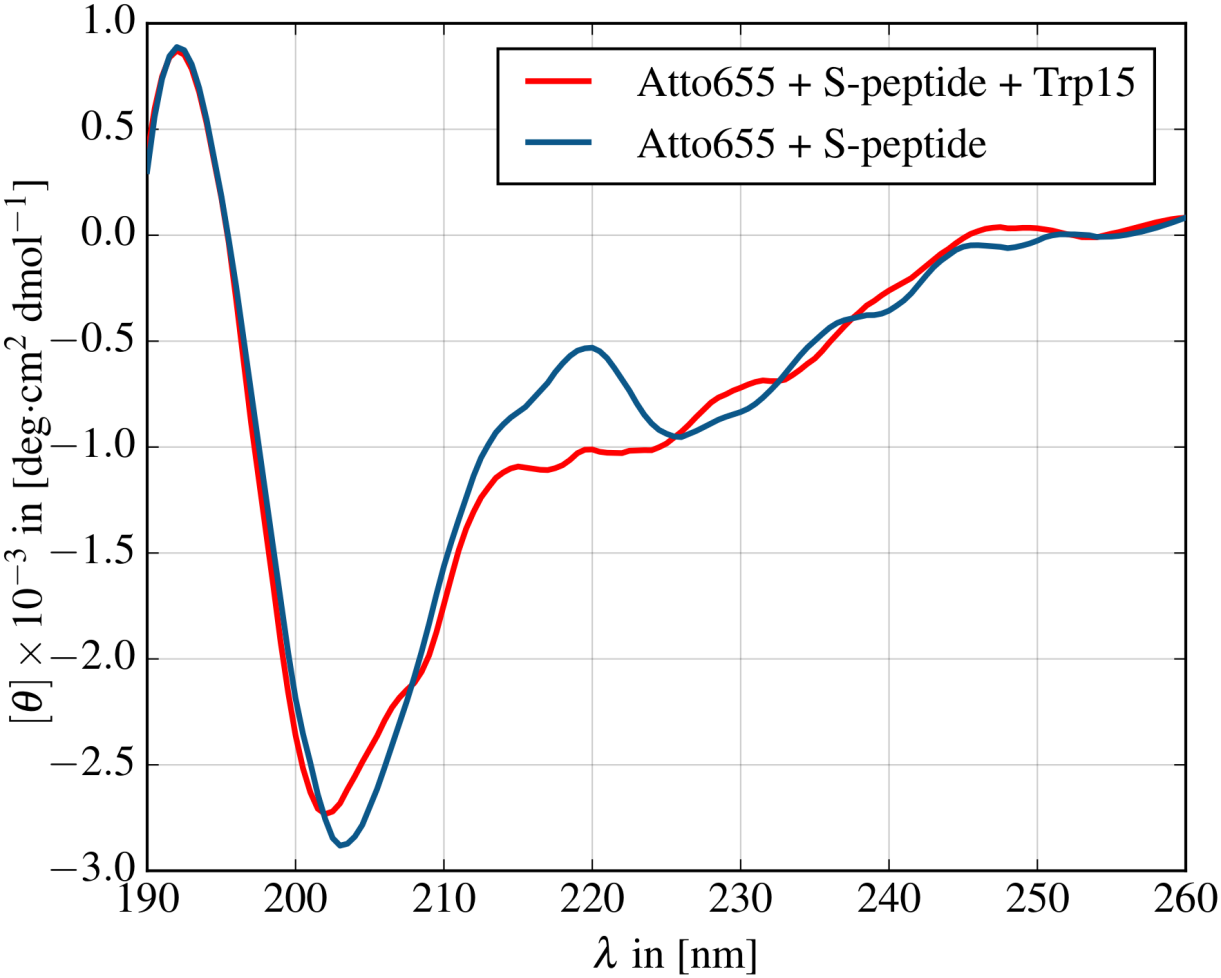
CD spectra of labeled S-peptide with and without Atto655 stacking partner Trp15. The peak at 220 nm indicates residual *α*-helix formation and less *β*-sheet contribution in the labeled S-peptide without Trp15.

The MD simulations suggest that the labeled S-peptide adopts a metastable *β*-sheet conformation induced by *π*-stacking interaction between the termini. To investigate to what extent the *β*-sheet conformation is part of the equilibrium regime and to quantify the modification in the accessible conformational regimes by dye/quencher interactions, we measured circular dichroism (CD) spectra of labeled S-peptide and compared it with the spectrum of S-peptide labeled with Atto655 but without Trp15 (Fig 3). Comparison with a previously published CD spectrum of native 20 residue S-peptide reveals qualitative similarity with the spectrum of labeled S-peptide without Trp15 where both spectra exhibit a local peak at 220 nm [36, 37]. When Trp15 is added to the peptide, however, the peak at 220 nm vanishes, consistent with the increase in *β*-sheet structure in our simulations, possibly induced by the *π*-stacking interaction between the terminal labels. The CD spectrum reveals that the relevant conformational regime is not dominated by only *β*-sheet structures but includes contributions of helical and random coil conformations. This supports the results from the 30 μs simulation where the labeled S-peptide adopted *β*-sheet conformation only during a fraction of the simulation time and exposed random coil backbone configurations otherwise (Fig 1, cluster #1).

### PET-FCS measurements

**Fig 4:**
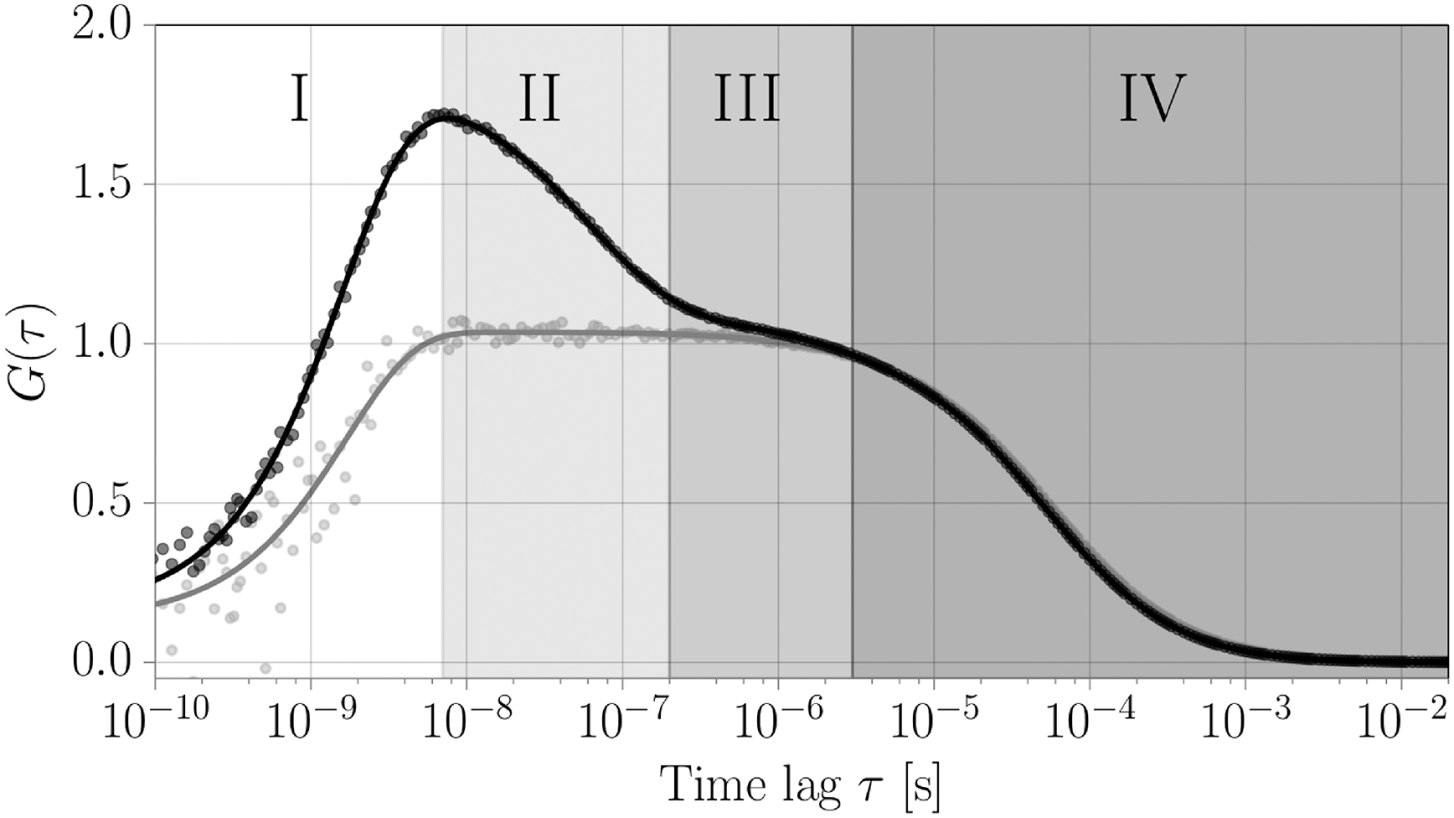
Experimentally obtained FCS curve and model fit function for labeled S-peptide in the presence (black) and absence (gray) of the quenching tryptophan. Indicated are the four main time regimes of the relevant processes. (I) Photon antibunching: The lifetime of the excited state of the fluorophore determines the shortest temporal separation between two photon emission events. This leads to a decrease in the correlation function for correlation times faster than the lifetime of the excited state. (II) Timescale of the internal conformational dynamics that lead to quenching/unquenching of the fluorophore and dominate the autocorrelation function. These timescales are to be compared with the MD simulations. (III) Photophysical artifacts: Intersystem crossing from the excited singlet state into a dark triplet state with lifetimes in the range of several μs (IV) This correlation regime is dominated by the diffusion of labeled peptides through the confined detection volume of the FCS setup.

The dye/quencher dynamics of the labeled S-peptide can be adressed experimentally by PET-FCS. The full PET-FCS correlation function is shown in Fig 4. Fluorescence correlation spectroscopy (FCS) analyzes the fluctuations of the recorded fluorescence signal and is thus sensitive to all processes affecting the fluorescence signal. Multiple phenomena spanning the timescale from picoseconds to seconds can be observed in the autocorrelation curve (regimes I-IV). Photon antibunching (I), a typical property of quantum emitters, occurs on the timescale of the fluorescence lifetime of ∼2 ns for Atto655 [38]. Diffusion through the confocal volume (IV) is observed on timescales between several tenths of μs to ms, depending on the size of the observation volume and the diffusion coefficient of the molecule. The measured diffusion time of the labeled S-peptide is 51 μs, translating to a diffusion coefficient of about 185 μm^2^s^−1^ (based on the lateral size of the focal volume of 195 nm), as is expected for a small peptide. Most fluorescent dyes can undergo intersystem crossing from the excited singlet state into a dark triplet state with lifetimes in the range of several μs (III). Here, we find a triplet relaxation time of 2.4 μs for Atto655. The amplitude of this triplet contribution is dependent on the incident laser power (Fig A in S1 File). Any conformational dynamics are superimposed onto these processes. The fast chain dynamics of intrinsically disordered peptides or unfolded proteins usually occur on the submicrosecond timescale [39], while slower conformational dynamics involving large conformational rearrangements usually take place in the range of ms to s [40].

**Fig 5:**
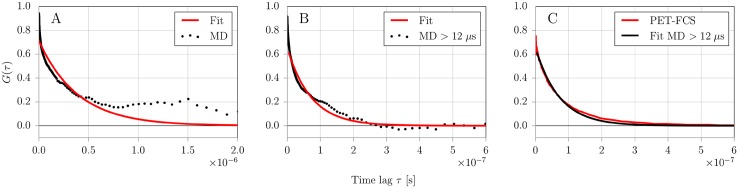
Atto655/Trp15 fluorescence quenching autocorrelation data fitted with a two-state exponential model function. Data and fits are shown for MD simulations (A, B) and experimental PET-FCS measurement (C). (A) Data from MD calculated over the whole simulation time (30 μs). (B) Data from MD where the initial 12 μs of the simulation was omitted. (C) Dynamic part of the correlation curve from experimental PET-FCS measurement (red) overlayed with the fitted MD data collected after 12 μs (black).

The quenching contribution to the correlation function is indicated in regime II (Fig 4). To determine the timescales of the quenching dynamics, we fit the correlation function with a model accounting for the listed contributions (Eq (3)). By careful inspection of the weighted residuals between model and data, we find that the addition of a second dynamic contribution to the model function improves the quality of the fit significantly (Fig B in S1 File and Table A in S1 File). Because the length of the MD simulation is not sufficient to address the existence of two dynamic contributions, however, we can not determine whether the second component arises from an alternative conformational regime of S-peptide. Therefore, we limit our discussion to the average timescale and overall amplitude of the two components. This result, however, indicates that the conformational dynamics may be more complex than discussed in this work. To directly compare the measured dynamics to the correlation functions obtained from MD, we isolate the dynamic contribution by dividing the correlation function by the contributions of diffusion, photophysics and antibunching (Fig 5B). We converted the observed amplitude of 0.67 and relaxation time of 80 ns to off- and on-rates by Eqs (4) and (5) yielding an off-rate *k*_off_ = 5.0 μs^−1^ and an on-rate *k*_on_ = 7.4 μs^−1^.

### Quenching dynamics from MD

The result obtained for the conformational dynamics of the S-peptide with labels based on the classification into “unfolded” and “folded” states by means of the end-to-end distance (2.7 μs^−1^) only roughly correlates with the rates of quenching contact formation as measured in the PET-FCS experiment (average rate of 6.2 μs^−1^). The quenching on- and off-rates describe the kinetics of quenching contact formation between the fluorophore Atto655 and the quencher Trp15, which are different from the conformational dynamics. To compare the experimental result with the simulations, a simple distance criterion between dye and quencher is applied.

Configurations of Atto655/Trp15 from simulation were classified as “dark” state when the distance between the geometric centers of their ring compounds was below a quenching distance of r* < 0.55 nm or as fluorescent otherwise [41–43]. The quenching autocorrelation function was fit to a two-state kinetic model with single exponential kinetics (Eq (1)). Due to the global conformational rearrangements of S-peptide during the initial 12 μs and the associated metastable states with lifetimes in the microsecond regime, the convergence of quenching autocorrelation data was insufficient. This is especially visible at large correlation times *τ* > 500 ns (Fig 5A). The long-lived metastable states however dominated the quenching dynamics resulting in relaxation timescales of *τ*_*r*_ = 391 ns (Table 1).

**Table 1:** Relaxation time scales (*τ*_*r*_) and amplitudes (*a*_*r*_) of the fluorescence autocorrelation fitted with a single exponential two-state model for the experimental PET-FCS measurement and MD simulations over the total simulation (MD all) and discounting the initital 12 μs. For the PET-FCS result, the sum of kinetic amplitudes and the average relaxation time of the two dynamic components are shown. Additionally, relaxation times and amplitudes have been converted to microscopic on- and off-rates of the related quenching process using the Formulas (4) and (5). The rates correspond well with the average opening and closing frequency of quenching contact formation between dye and quencher in labeled S-peptide observed in the MD simulation.

	*a*_*r*_	*τ*_*r*_ [ns]	*k*_on_ [*μ*s^−1^]	*k*_off_ [*μ*s^−1^]
PET-FCS	0.67	80.4	7.4	5.0
MD >12 *μ*s	0.65	72.1	8.4	5.5
MD all	0.71	391.3	1.5	1.1

**Fig 6:**
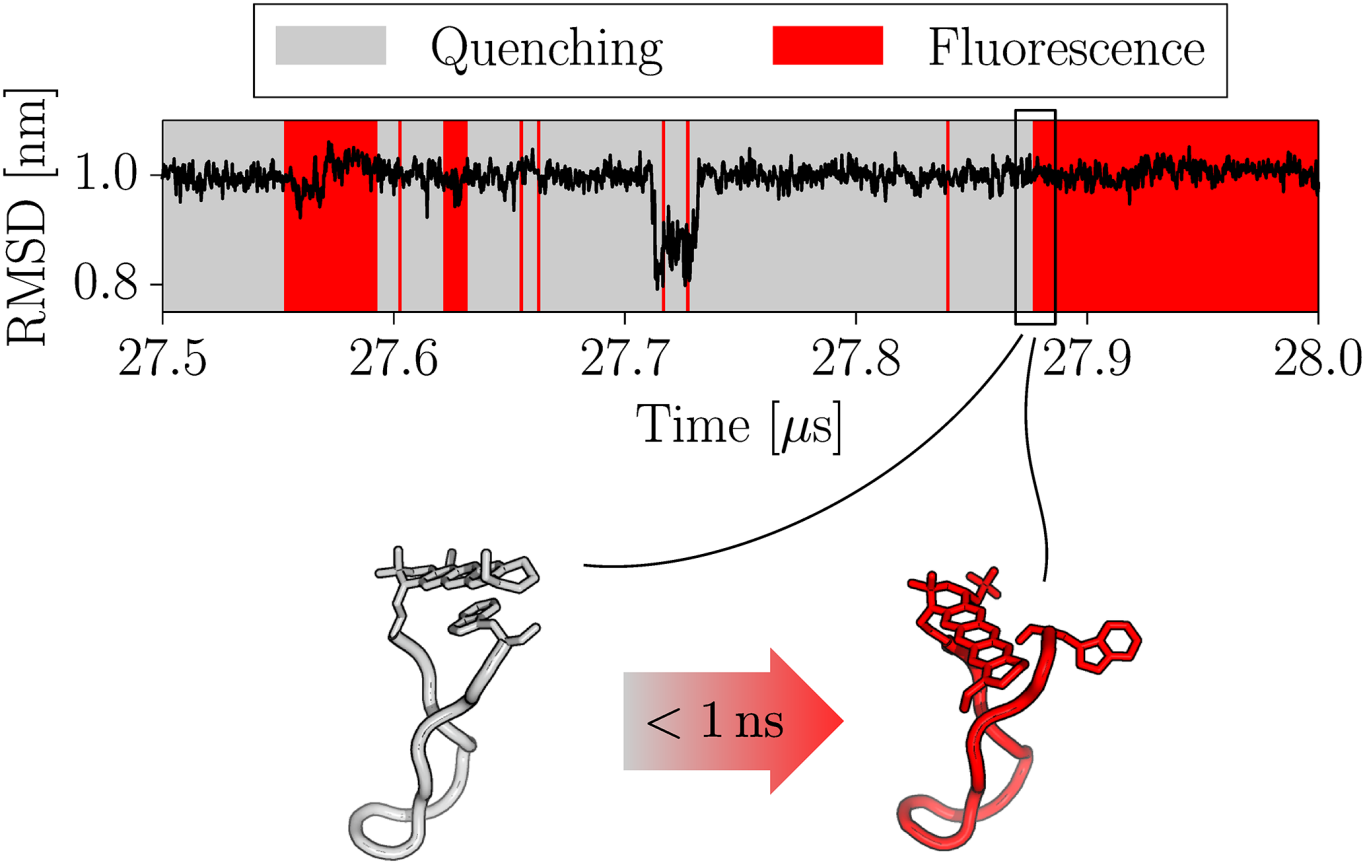
Time evolution of non-hydrogen atoms RMSD of residues 1-14 with respect to the unfolded starting structure and classification into quenched and fluorescent states in the time frame between 27.5–28.0 μs. Although the backbone was mostly locked in cluster #1 conformation during this time span, spontaneous unstacking of Atto655/Trp15 was observed. Two exemplary structures shortly before and after an unstacking event are shown below. The stacked (grey) configuration quenches the Atto655 fluorescence, while the unstacked (red) configuration allows fluorescence. Unstacking was observed to occur on the subnanosecond timescale and is not coupled to a noticable change of the overall RMSD in this cluster.

Because the S-peptide locked in a quasi-stable folded *β*–sheet like configuration after 12 μs simulation time, we decided to treat the initial 12 μs as equilibration time and recalculate the quenching autocorrelation for only the second part of the simulation (Fig 5B). The resulting quenching relaxation timescale of *τ*_*r*_ = 72 ns was about 4–5 times faster as dye and quencher could not diffuse far away from each other by the confined *β*-sheet like backbone structure. In the investigated simulation time window (12–30 μs), no global backbone rearrangements of S-peptide were observed and lifetimes of quenching states of the fluorescence labels were found in the range of hundreds of nanoseconds (see Fig 6). Omitting the initial 12 μs as equilibration also led to a significantly better agreement between the single exponential fit model and the simulation data especially for long relaxation times *τ*_*r*_. Similar to PET-FCS data treatment, we also calculated the on- and off-rates by Eqs (4) and (5) from the correlation amplitude and relaxation time. Comparative data between experiment and simulation for fitted relaxation parameters and rates are shown in Table 1. We find that the quenching dynamics for simulation data after 12 μs are in good agreement with the experimental results of 80 ns.

## Discussion

The interpretation of fluorescence spectroscopy measurements depends on the assumption that the artificial attachment of fluorophores does not alter the conformations and dynamics of the target molecule itself. To address this problem, one should whenever possible ensure the functionality of the labeled molecule e.g. by biochemical assays probing specific interactions or ATP turnover rates whenever possible. If such a test is not available, MD can provide important insight with respect to the influence of the fluorescence labels. We performed comparative MD simulations and PET-FCS measurements on fluorescently labeled 14 residue S-peptide, serving as a model system of an intrinsically disordered peptide. Our results reveal that the labeling strongly affects both the conformational and dynamical properties of the S-peptide, shifting it from the disordered conformational regime to a semi-stable fold with *β*-sheet content. This drastic change is caused mainly by the strong *π*-stacking interaction between the rigid ring systems of Atto655 and Trp15, which traps the termini of the peptide to remain in close vicinity. This effect may occur with many widely used fluorophores containing rigid ring systems as their light absorbing centers that facilitate stacking interactions with other aromatic ring structures.

The agreement between quenching correlation functions from MD simulations and PET-FCS measurement is surprisingly good, although only when skipping the initial 12 μs of the MD trajectory as equilibration time (Table 1). As the labeled S-peptide was started from an arbitrary extended starting structure, we assumed that the system requires some time to reach equilibrium and treated the initial 12 μs as equilibration time, thereby dividing the dynamic S-peptide regime in two characteristic regimes. The first part of the simulation was dominated by global backbone rearrangements and slow quenching dynamics as the peptide backbone continuously stretched and refolded. During the second part of the simulation, the S-peptide backbone locked into a meta-stable fold with only the label side chains stacking and unstacking from time to time. Simulation time, however, is finite, therefore it remains unclear to what degrees these two regimes contribute to the equilibrium regime. As we reproduce good agreement between simulation and experiment for the second regime with faster quenching dynamics, we assume that S-peptide preferably resides in this regime. However, the fit quality of experimental PET-FCS data can be improved by adding an additional dynamic term with a slower relaxation coefficient (Fig B in S1 File). We speculate, that this second dynamic term might correspond to the regime of global rearrangements seen in the initial part of MD simulations. It is interesting to note, that the PET-FCS method does not necessarily detect global structural dynamics. As long as the global dynamics are not strongly coupled to the local dynamics of dye and quencher, they might remain undetected in the experiment. Surprisingly, such a scenario is possible even for short sequences as of the S-peptide. Our simulations reveal, that stacking/unstacking dynamics occuring simultaneously with a stable backbone configuration in one regime are difficult to distinguish with solely experimental data from another regime with large conformational backbone rearrangements (Fig 6).

The good agreement between experiment and simulation is even more surprising as the quenching state is judged only by a simple distance criterion, which was proposed earlier by Vaiana et. al [41] and was based on a fit of fluorophore MD trajectories to correlation spectroscopy data. Previous studies successfully demonstrated that the connection between atomistic MD simulations and experimental PET-FCS measurements can be drawn with such a simple criterion [42]. It should be noted that effects evolving from the photoinduced charge separation among Atto655 and Trp15 are not included in the approach as the MD parameters represent only the neutral state. We speculate that the opposite charges could increase the interaction between the dyes due to the coulomb attraction, a question that could in principle be targeted by measurements at different salt concentrations. Gaining a more detailed understanding of the relation between dye/quencher orientation and fluorescence quenching would require to derive redox potentials for single MD frames via quantum chemical calculations. Here, we use the experimental PET-FCS measurements for the validation of our labeled S-peptide simulations, whereas only the comparison between simulations with and without labels reveal the stark influence of fluorescence labeling on the dynamics and sampled conformational regimes of the peptide. The circular dichroism measurements of labeled and unlabeled S-peptide strongly support these findings.

When using fluorescence to study biomolecules, two major types of artifacts need to be considered. On the one hand, the properties of the fluorophore may be altered by the local environment of the biomolecule, changing the quantum yield, leading to spectral shifts of emission or absorption spectra or hindering the rotational motion through sterical hindrance or specific interactions. Our study, on the other hand, sheds light on the effect of the chemical modification on the target biomolecule and shows that the fluorescence label can significantly affect the conformational and dynamical properties. In particular, PET-FCS experiments, which intrinsically require that the fluorophore and quencher can come into close contact, suffer from possible interactions between them. The influence of specific dye-quencher or dye-dye interactions on the conformational dynamics is especially pronounced when investigating intrinsically disordered systems as presented here, for which PET and FRET are often the method of choice. Although the effect of fluorescence labeling on the dynamics and conformations of the peptide in this study was found to be significant, other systems might be affected to different degrees. Combined MD and FRET studies of labeled polyproline for example, found only little impact of the fluorophores on the sampled conformational regimes. However, polyproline is characteristic for its rigid structure with deep energy minima and slow trans/cis isomerisation dynamics on timescales beyond minutes, which were not covered directly by that study [27]. Nevertheless, it is very likely that especially small peptides with a large degree of intrinsic disorder and a relatively flat energy landscape suffer most from fluorescence labeling artifacts. For larger and more stable proteins with clear conformational preferences, on the other hand, we expect the artifacts induced by the fluorophores to be of reduced importance.

In summary, great care needs to be taken when designing and interpreting fluorescence experiments. Especially when fluorescence labeling is used to study systems for which the size of the dye is comparable to the target molecule, a single dye interaction can dominate the conformational properties and severely effect the results. The position for the fluorescent label should be chosen in a well-structured and solvent-exposed part of the protein sequence to minimize the influence of the fluorophore on the local structure and dynamics, especially when partly disordered proteins are studied. We demonstrate that MD is a valuable tool that complements PET-FCS experiments by offering an atomistic interpretation of the experimental result. Furthermore, simulations provide a useful strategy to predict the influence of labeling configurations on systematic properties when designing fluorescence experiments. On the other hand, fluorescence experiments prove useful to validate *in silico* results by providing a reference for dynamic parameters of the system.

## Methods

### Molecular dynamics

Two simulations of the S-peptide, one with and the other without labels, were started from extended peptide conformations. The periodic box boundaries were set at a minimal distance of 1 nm to the peptide. The peptide was parametrized with the Amber99sb-ILDN forcefield [44]. Parameters for the Atto655 fluorophore were generated as follows. First two conformations of the fluorophore were geometry optimized at *ab initio* B3LYP/6-31G* level using the Gaussian09 software [45–48]. Second the partial charges were generated by fitting the electrostatic potential derived from HF/6-31G* quantum mechanical calculations with the restraint electrostatic potential protocol (RESP) [49]. Finally, atom types and bonded/nonbonded parameters were assigned from the general amber forcefield (GAFF) [50]. The parametrization protocol followed the guidelines of GAFF which was designed to be compatible with the Amber forcefields. Both systems were energy minimized and equilibrated in NPT ensemble (298 K, 1.01 bar) after the addition of explicit solvent Tip3P water molecules [51]. Respectively four positive and negative counter ions were added to the solution keeping the box charge neutral. The production run was performed at a time step of 3 fs with the GROMACS 5.0 MD software suite [52, 53] using the velocity rescaling thermostat in the NVT ensemble (298 K) [54]. Bonds involving hydrogens were constrained with the LINCS algorithm [55].

### Peptide synthesis

The 14 amino acid truncated S-peptide (KETAA AKFER QHMD) was used as a model system for an intrinsically disordered peptide [28–31]. To fluorescently label the S-peptide for PET-FCS measurements, the fluorophore Atto655 was attached to the N terminus and an additional tryptophan was added to the C terminus serving as a fluorescence quencher [3]. The resulting sequence of the labeled S-peptide was Atto655 KETAA AKFER QHMDW. Peptide synthesis, labeling and purification was performed as described previously [56].

### PET-FCS measurements

PET-FCS measurements were performed on a custom-built confocal single-molecule fluorescence microscope. The sample was excited with a diode laser (LDH-D-C-640, PicoQuant) operated in continuous wave mode at an average laser power of 280 μW as measured before the aperture of the objective. The fluorescence signal was passed through a pinhole and split on two avalanche photodiodes (SPCM-AQR-14, Perkin Elmer Optoelectronics) by a 50:50 beam splitter to avoid detector dead time, connected to two independent channels of the time-correlated single photon counting (TCSPC) hardware (HydraHarp400, PicoQuant). Fluorescence signal was passed through an emission filter (ET670/30, AHF Analysentechnik). The range of the emission filter was chosen as to avoid detector crosstalk due to the breakdown flash of the APDs [57]. Individual photon arrival times were recorded with 16 ps resolution. S-peptide was dissolved in standard PBS buffer with 0.005% Tween-20 to prevent sticking to the cover slide surface, and diluted to a final concentration of ∼1 nM. FCS data was collected over a time of 16 h at room temperature.

### Quenching autocorrelation

Configurations of Atto655/Trp15 from simulation were classified as “dark” state when the distance between the geometric centers of their ring compounds was below the quenching distance of r* < 0.55 nm, or as fully fluorescent otherwise [41–43]. To calculate the quenching autocorrelation function, 10^6^ frames from the 30 μs MD trajectory were analyzed. The quenching signal autocorrelation data was fitted with a two-state model with a single exponential signal decay

$$\begin{array}{c} G_{\text{dyn}}\left(\tau \right)=a_{r}e^{-\tau /\tau_{r}} \end{array}$$ (1)

where *a*_*r*_ is the amplitude and *τ*_*r*_ the relaxation time constant of the quenching relaxation autocorrelation.

### FCS data analysis

The second order intensity cross-correlation function *G*_*ij*_(*τ*) between two channels *i* and *j* is defined by:

$$\begin{array}{c} G_{ij}\left(\tau \right)=\frac{\left\langle I_{i}\left(t\right)I_{j}\left(t+\tau \right)\right\rangle }{\left\langle I_{i}\left(t\right)\right\rangle \left\langle I_{j}\left(t\right)\right\rangle } \end{array}$$ (2)

where *I*_*i*_(*t*) is the intensity in channel *i* at time *t*, *τ* is the time lag and 〈〉 denotes time averaging. Cross-correlation functions between the two detectors were computed using custom-written software based on a multiple-tau correlation algorithm [58]. Error bars are determined by splitting the measurement into ten segments of equal length and computing the standard error of mean of the correlation functions. Fitting of the correlation function was performed in MATLAB (The Mathworks, Inc.) using the non-linear least squares fit routine by minimizing the weighted residuals. Confidence intervals (95%) of determined parameters are computed from the covariance matrix obtained from the fit procedure. FCS curves are fit using a model accounting for photon antibunching, triplet kinetics and diffusion, as well as one or two additional bunching terms for the observed kinetics:

$$\begin{array}{ccc} G(\tau ) & = & \frac{\gamma }{N}{\left(1+\frac{\tau }{\tau_{D}}\right)}^{-1}{\left(1+\frac{\tau }{p^{2}\tau_{D}}\right)}^{-1/2}\times \\ & & \left(1-A_{ab}e^{-\tau /\tau_{ab}}\right)\left(1+\frac{T}{1-T}e^{\frac{\tau }{\tau_{T}}}\right)\times \\ & & \left(1+a_{r,1}e^{-\tau /\tau_{r,1}}+a_{r,2}e^{-\tau /\tau_{r,2}}\right) \end{array}$$ (3)

where *a*_*r*,1/2_ are the amplitudes and *τ*_*r*,1/2_ the relaxation time constants of the quenching relaxation autocorrelation [20], *τ*_*D*_ is the diffusion time constant, *N* is the average number of particles in the focus, *γ* = 2^−3/2^ is a correction factor accounting for the shape of the confocal volume, *p* is the ratio of axial to lateral size of the confocal volume, *τ*_*T*_ is the triplet time constant, *T* is the triplet fraction, and *τ*_*ab*_ and *A*_*ab*_ are the photon antibunching amplitude and relaxation time. The parameters of the dynamic quenching term are related to the off- and on-rates of the quenching process by [59]:

$$\begin{array}{c} \tau_{r}=\frac{1}{k_{\text{on}}+k_{\text{off}}} \end{array}$$ (4)

$$\begin{array}{c} a_{r}=\frac{k_{\text{off}}}{k_{\text{on}}} \end{array}$$ (5)

In terms of the system at hand, *k*_on_ and *k*_off_ can be interpreted as the microscopic rate constants of end-to-end contact formation and dissociation.

### Circular dichroism

CD measurements were performed on a Jasco J-715 spectrophotometer at 25°C. Labeled S-peptide with and without Trp15 was solvated at 1 mg ml^−1^ concentration in DPBS buffer (Sigma-Aldrich). The samples were measured in a quartz cell with 0.2 mm path length. Data is expressed in terms of mean residue molecular elliptisity [*θ*]_λ_. CD data was smoothed using a Savitzky-Golay-Filter with order 3 and a window of 10 nm [60].

## Supporting information

S1 File The supporting information gives details on the PET-FVS dynamics, control experiments and results on the PET-FCS measurements. **Fig A, PET-FCS dynamics.** Fit of the experimental correlation function using a model accounting for one kinetic component and two kinetic components. **Fig B, Control experiments. Table A, Fit results for PET-FCS measurement.**
(PDF)

## References

[1] Kiefhaber T, Bachmann A, Jensen KS. Dynamics and mechanisms of coupled protein folding and binding reactions. Current Opinion in Structural Biology. 2012;22(1):21–29. 10.1016/j.sbi.2011.09.010 22129832

[2] Neuweiler H, Sauer M. Using photoinduced charge transfer reactions to study conformational dynamics of biopolymers at the single-molecule level. Current pharmaceutical biotechnology. 2004;5(3):285–298. 10.2174/1389201043376896 15180550

[3] Doose S, Neuweiler H, Sauer M. Fluorescence Quenching by Photoinduced Electron Transfer: A Reporter for Conformational Dynamics of Macromolecules. ChemPhysChem. 2009;10(9-10):1389–1398. 10.1002/cphc.200900238 19475638

[4] Jares-Erijman EA, Jovin TM. FRET imaging. Nature biotechnology. 2003;21(11):1387–1395. 10.1038/nbt896 14595367

[5] Weiss S. Fluorescence spectroscopy of single biomolecules. Science. 1999;283(5408):1676–1683. 10.1126/science.283.5408.1676 10073925

[6] Ha T. Single-Molecule Fluorescence Resonance Energy Transfer. Methods. 2001;25(1):78–86. 10.1006/meth.2001.1217 11558999

[7] Förster T. Zwischenmolekulare Energiewanderung und Fluoreszenz. Annalen der physik. 1948;437(1-2):55–75. 10.1002/andp.19484370105

[8] Kavarnos GJ. Fundamentals of photoinduced electron transfer. vol. 98 Wiley-VCH; 1994.

[9] Kavarnos GJ, Turro NJ. Photosensitization by reversible electron transfer: theories, experimental evidence, and examples. Chemical Reviews. 1986;86(2):401–449. 10.1021/cr00072a005

[10] Weller A. Photoinduced electron transfer in solution: exciplex and radical ion pair formation free enthalpies and their solvent dependence. Zeitschrift für Physikalische Chemie. 1982;.

[11] de Silva AP, Gunaratne HQN, Gunnlaugsson T, Huxley AJM, McCoy CP, Rademacher JT, et al Signaling Recognition Events with Fluorescent Sensors and Switches. Chemical Reviews. 1997;97(5):1515–1566. 10.1021/cr960386p 11851458

[12] Zhong D, Pal SK, Wan C, Zewail AH. Femtosecond dynamics of a drug–protein complex: daunomycin with Apo riboflavin-binding protein. Proceedings of the National Academy of Sciences. 2001;98(21):11873–11878. 10.1073/pnas.211440298 PMC59816 11592998

[13] Li X, Zhu R, Yu A, Zhao XS. Ultrafast photoinduced electron transfer between tetramethylrhodamine and guanosine in aqueous solution. The Journal of Physical Chemistry B. 2011;115(19):6265–6271. 10.1021/jp200455b 21491918

[14] Michalet X, Kapanidis AN, Laurence T, Pinaud F, Doose S, Pflughoefft M, et al The power and prospects of fluorescence microscopies and spectroscopies. Annual review of biophysics and biomolecular structure. 2003;32(1):161–182. 10.1146/annurev.biophys.32.110601.142525 12598370

[15] Tinnefeld P, Sauer M. Branching out of single-molecule fluorescence spectroscopy: Challenges for chemistry and influence on biology. Angewandte Chemie International Edition. 2005;44(18):2642–2671. 10.1002/anie.200590060 15849689

[16] Elson EL, Magde D. Fluorescence correlation spectroscopy. I. Conceptual basis and theory. Biopolymers. 1974;13(1):1–27. 10.1002/bip.1974.360130102 4818131

[17] Elson EL. Fluorescence Correlation Spectroscopy: Past, Present, Future. Biophysical Journal. 2011;101(12):2855–2870. 10.1016/j.bpj.2011.11.012 22208184 PMC3244056

[18] Sahoo H, Schwille P. FRET and FCS—Friends or Foes? ChemPhysChem. 2011;12(3):532–541. 10.1002/cphc.201000776 21308943

[19] Felekyan S, Kalinin S, Sanabria H, Valeri A, Seidel CAM. Filtered FCS: Species Auto- and Cross-Correlation Functions Highlight Binding and Dynamics in Biomolecules. ChemPhysChem. 2012;13(4):1036–1053. 10.1002/cphc.201100897 22407544 PMC3495305

[20] Sauer M, Neuweiler H. PET-FCS: probing rapid structural fluctuations of proteins and nucleic acids by single-molecule fluorescence quenching. Fluorescence Spectroscopy and Microscopy: Methods and Protocols. 2014; p. 597–615. 10.1007/978-1-62703-649-8_27 24108646

[21] Zhang Y, Yuan S, Lu R, Yu A. Ultrafast fluorescence quenching dynamics of Atto655 in the presence of N-acetyltyrosine and N-acetyltryptophan in aqueous solution: proton-coupled electron …. The Journal of Physical Chemistry. 2013;117(24):7308–7316. 10.1021/jp404466f 23721323

[22] Milles S, Tyagi S, Banterle N, Koehler C, VanDelinder V, Plass T, et al Click Strategies for Single-Molecule Protein Fluorescence. Journal of the American Chemical Society. 2012;134(11):5187–5195. 10.1021/ja210587q 22356317

[23] Haustein E, Schwille P. Fluorescence correlation spectroscopy: novel variations of an established technique. Annu Rev Biophys Biomol Struct. 2007;36:151–169. 10.1146/annurev.biophys.36.040306.132612 17477838

[24] Moerner W, Fromm DP. Methods of single-molecule fluorescence spectroscopy and microscopy. Review of Scientific Instruments. 2003;74(8):3597–3619. 10.1063/1.1589587

[25] Daidone I, Neuweiler H, Doose S, Sauer M, Smith JC. Hydrogen-bond driven loop-closure kinetics in unfolded polypeptide chains. PLoS Computational Biology. 2010;6(1):e1000645 10.1371/journal.pcbi.1000645 20098498 PMC2799665

[26] Schröder GF, Alexiev U, Grubmüller H. Simulation of fluorescence anisotropy experiments: probing protein dynamics. Biophysical Journal. 2005;89(6):3757–3770. 10.1529/biophysj.105.069500 16169987 PMC1366944

[27] Hoefling M, Lima N, Haenni D, Seidel CAM, Schuler B, Grubmüller H. Structural Heterogeneity and Quantitative FRET Efficiency Distributions of Polyprolines through a Hybrid Atomistic Simulation and Monte Carlo Approach. PLoS ONE. 2011;6(5):e19791 10.1371/journal.pone.0019791 21629703 PMC3101224

[28] Finn F, Dadok J, Bothner-By A. Proton nuclear magnetic resonance studies of the association of ribonuclease S-peptide and analogs with ribonuclease S-protein. Biochemistry. 1972;11(3):455–461. 10.1021/bi00753a025 5062062

[29] Bastos M, Pease JH, Wemmer DE, Murphy KP, Connelly PR. Thermodynamics of the helix-coil transition: Binding of S15 and a hybrid sequence, disulfide stabilized peptide to the S-protein. Proteins: Structure, Function, and Bioinformatics. 2001;42(4):523–530. 10.1002/1097-0134(20010301)42:4<523::AID-PROT100>3.0.CO;2-B 11170206

[30] Cole R, Loria JP. Evidence for flexibility in the function of ribonuclease A. Biochemistry. 2002;41(19):6072–6081. 10.1021/bi025655m 11994002

[31] Marshall GR, Feng JA, Kuster DJ. Back to the future: ribonuclease A. Peptide Science. 2008;90(3):259–277. 10.1002/bip.20845 17868092

[32] Bachmann A, Wildemann D, Praetorius F, Fischer G, Kiefhaber T. Mapping backbone and side-chain interactions in the transition state of a coupled protein folding and binding reaction. Proceedings of the National Academy of Sciences. 2011;108(10):3952–3957. 10.1073/pnas.1012668108 PMC3054012 21325613

[33] Richards FM. ON THE ENZYMIC ACTIVITY OF SUBTILISIN-MODIFIED RIBONUCLEASE. Proceedings of the National Academy of Sciences of the United States of America. 1958;44:162–166. 10.1073/pnas.44.2.162 16590160 PMC335382

[34] Richards FM, Vithayathil PJ. The Preparation of Subtilisin-modified Ribonuclease and the Separation of the Peptide and Protein Components. Journal of Biological Chemistry. 1959;234:1459–1465. 13654398

[35] Murtagh F, Contreras P. Algorithms for hierarchical clustering: an overview. Wiley Interdisciplinary Reviews: Data Mining and Knowledge Discovery. 2012;2(1):86–97.

[36] Simons ER, Blout ER. Circular Dichroism of Ribonuclease A, Ribonuclease S, and Some Fragments. The Journal of biological chemistry. 1968;243(1):218–221. 5635944

[37] Tamburro A, Scatturin A, Rocchi R, Marchiori F, Borin G, Scoffone E. Conformational-transitions of bovine pancreatic ribonuclease S-peptide. FEBS letters. 1968;1(5):298–300. 10.1016/0014-5793(68)80137-1 11945325

[38] Mets Ü, Widengren J, Rigler R. Application of the antibunching in dye fluorescence: measuringthe excitation rates in solution. Chemical Physics. 1997;.

[39] Nettels D, Gopich IV, Hoffmann A, Schuler B. Ultrafast dynamics of protein collapse from single-molecule photon statistics. Proceedings of the National Academy of Sciences of the United States of America. 2007;104(8):2655–2660. 10.1073/pnas.0611093104 17301233 PMC1815237

[40] Weiss S. Measuring conformational dynamics of biomolecules by single molecule fluorescence spectroscopy. Nature Structural Biology. 2000;. 10.1038/78941 10966638

[41] Vaiana AC, Neuweiler H, Schulz A, Wolfrum J, Sauer M, Smith JC. Fluorescence quenching of dyes by tryptophan: interactions at atomic detail from combination of experiment and computer simulation. Journal of the American Chemical Society. 2003;125(47):14564–14572. 10.1021/ja036082j 14624606

[42] Noé F, Doose S, Daidone I, Löllmann M, Sauer M, Chodera JD, et al Dynamical fingerprints for probing individual relaxation processes in biomolecular dynamics with simulations and kinetic experiments. Proceedings of the National Academy of Sciences. 2011;108(12):4822–4827. 10.1073/pnas.1004646108 PMC3064371 21368203

[43] Kalinin S, Peulen T, Sindbert S, Rothwell PJ, Berger S, Restle T, et al A toolkit and benchmark study for FRET-restrained high-precision structural modeling. Nature Methods. 2012;9(12):1218–1225. 10.1038/nmeth.2222 23142871

[44] Lindorff-Larsen K, Piana S, Palmo K, Maragakis P, Klepeis JL, Dror RO, et al Improved side-chain torsion potentials for the Amber ff99SB protein force field. Proteins. 2010;78(8):1950–1958. 10.1002/prot.22711 20408171 PMC2970904

[45] Frisch MJ, Trucks GW, Schlegel HB, Scuseria GE, Robb MA, Cheeseman JR, et al.. Gaussian 09 Revision D.01;.

[46] Becke AD. Density-functional exchange-energy approximation with correct asymptotic behavior. Phys Rev A. 1988;38:3098–3100. 10.1103/PhysRevA.38.3098 9900728

[47] Lee C, Yang W, Parr RG. Development of the Colle-Salvetti correlation-energy formula into a functional of the electron density. Phys Rev B. 1988;37:785–789. 10.1103/PhysRevB.37.785 9944570

[48] Hariharan PC, Pople JA. The influence of polarization functions on molecular orbital hydrogenation energies. Theor Chim Acta. 1973;28(3):213–222. 10.1007/BF00533485

[49] Bayly CI, Cieplak P, Cornell WD, Kollman PA. A Well-Behaved Electrostatic Potential Based Method Using Charge Restraints for Deriving Atomic Charges: The RESP Model. J Phys Chem. 1993;97(40):10269–10280. 10.1021/j100142a004

[50] Wang J, Wolf RM, Caldwell JW, Kollman PA, Case DA. Development and testing of a general amber force field. J Comput Chem. 2004;25(9):1157–1174. 10.1002/jcc.20035 15116359

[51] Jorgensen WL, Chandrasekhar J, Madura JD, Impey RW, Klein ML. Comparison of simple potential functions for simulating liquid water. J Chem Phys. 1983;79(2):926–935. 10.1063/1.445869

[52] Hess B, Kutzner C, Spoel DVD, Lindahl E. GROMACS 4: Algorithms for highly efficient, load-balanced, and scalable molecular simulation. J Chem Theory Comput. 2008;4:435–447. 10.1021/ct700301q 26620784

[53] Van Der Spoel D, Lindahl E, Hess B, Groenhof G, Mark AE, Berendsen HJC. GROMACS: Fast, flexible, and free. J Comput Chem. 2005;26(16):1701–1718. 10.1002/jcc.20291 16211538

[54] Bussi G, Donadio D, Parrinello M. Canonical sampling through velocity rescaling. The Journal of chemical physics. 2007;126(1):014101 10.1063/1.2408420 17212484

[55] Hess B, Bekker H, Berendsen HJC, Fraaije JGEM. LINCS: A linear constraint solver for molecular simulations. Journal of Computational Chemistry. 1997;18(12):1463–1472. 10.1002/(SICI)1096-987X(199709)18:12<1463::AID-JCC4>3.0.CO;2-H

[56] Crevenna AH, Naredi-Rainer N, Lamb DC, Wedlich-Söldner R, Dzubiella J. Effects of Hofmeister Ions on the a-Helical Structure of Proteins. Biophysical Journal. 2012;102(4):907–915. 10.1016/j.bpj.2012.01.035 22385862 PMC3283803

[57] Kurtsiefer C, Zarda P, Mayer S, Weinfurter H. The breakdown flash of Silicon Avalance Photodiodes—backdoor for eavesdropper attacks? arXivorg. 2001;(13):2039–2047.

[58] Felekyan S, Kühnemuth R, Kudryavtsev V, Sandhagen C, Becker W, Seidel CAM. Full correlation from picoseconds to seconds by time-resolved and time-correlated single photon detection. Review of Scientific Instruments. 2005;76(8):083104 10.1063/1.1946088

[59] Neuweiler H, Johnson CM, Fersht AR. Direct observation of ultrafast folding and denatured state dynamics in single protein molecules. Proceedings of the National Academy of Sciences. 2009;106(44):18569–18574. 10.1073/pnas.0910860106 PMC2773960 19841261

[60] Greenfield NJ. Using circular dichroism spectra to estimate protein secondary structure. Nature Protocols. 2006;1(6):2876–2890. 10.1038/nprot.2006.202 17406547 PMC2728378

