# Supplementary material for: Covalent dye attachment influences the dynamics and conformational properties of flexible peptides
Source: PLoS One. 2017 May 23;12(5):e0177139. doi: 10.1371/journal.pone.0177139 (PMC5441599; doi:10.1371/journal.pone.0177139)
Supplement: S1 File — Fig A, PET-FCS dynamics. Fit of the experimental correlation function using a model accounting for one kinetic component and two kinetic components. Fig B, Control experiments. Table A, Fit results for PET-FCS measurement. (PDF) [file pone.0177139.s001.pdf]

Supporting Information: Covalent dye attachment influences the dynamics and conformational properties of flexible peptides

Manuel P. Luitz,* Anders Barth,* Alvaro H. Crevenna,

Rainer Bomblies, Don C. Lamb, and Martin Zacharias†

Department Physik, T38, Technische Universität München, 85748 Garching, Germany‡

Department Chemie, Physikalische Chemie,

Ludwig-Maximilians-Universität München, 81377 München, Germany and

Instituto de Tecnologia Química e Biológica António Xavier,

Universidade Nova de Lisboa, 2780-157 Oeiras, Portugal

(Dated: April 11, 2017)

* M. P. Luitz and A. Barth contributed equally to this work

† mzacharias@mytum.de

‡ http://www.t38.ph.tum.de

SUPPLEMENTARY EXPERIMENTAL RESULTS

Control measurements

We characterized the labeled S-peptide in terms of fluorescence lifetime and anisotropy with respect to a control construct without a tryptophan (Figure B A and B). The fluorescence lifetime of Atto655 changed slightly upon attachment to the peptide from 1.8 ns for the free dye (data not shown) to 2.18 ns. Upon addition of the tryptophan, the lifetime decreased slightly to 2.05 ns, caused by dynamic quenching on the timescale of the fluorescence lifetime[S1]. Using the Stern-Volmer equation[S2], the dynamic quenching rate k_q can be calculated from the quenched and unquenched lifetime τ_q and τ_u as:

$$k_q = \tau_q^{-1} - \tau_u^{-1} \tag{A}$$

This yields a relaxation time for the dynamic quenching process of ∼35 ns, in very good agreement with the observed fast quenching constant. No significant change in the fluorescence anisotropy was observed upon addition of the tryptophan. Only the unquenched fluorophores contribute to the anisotropy signal, thus it can be concluded that the rotational freedom of the fluorophores not engaged in π-stacking interactions with the quencher is not affected by the change in conformational dynamics observed in the simluations.

To indentify which observed kinetic amplitude is caused by photophysics, we performed a series of measurements at different excitation powers (Figure B D-F). The only significant change in the obtained correlation functions was the triplet amplitude (see Figure B F and inset in Figure B D), whereas the other kinetic amplitudes showed no power dependence (Figure B E).

[S1] S. Doose, H. Neuweiler, and M. Sauer, "Fluorescence quenching by photoinduced electron transfer: A reporter for conformational dynamics of macromolecules," *ChemPhysChem*, vol. 10, no. 9-10, pp. 1389–1398, 2009.

[S2] J. R. Lakowicz, *Principles of Fluorescence Spectroscopy.* Springer Science & Business Media, Dec. 2007.

TABLE A. Fit results for PET-FCS measurement. Errors are given as 95% confidence intervals. $\tau_{r,1,2}$ and $a_{r,1}$: relaxation times and amplitudes of the two dynamic terms, N: number of particles in the focal volume, τ_D: diffusion time, p: geometric parameter of the focal volume, τ_{ab} and A_{ab}: antibunching timescale and amplitude, τ_T and T: triplet relaxation time and fraction.

$\tau_{r,1}$	$a_{r,1}$	$\tau_{r,2}$	$a_{r,2}$
37 ± 5 ns	0.32 ± 0.05	120 ± 11 ns	0.35 ± 0.05

N	τ_D	p	τ_{ab}	A_{ab}	τ_T	T
0.411 ± 0.001	51.4 ± 0.2 μs	4.28 ± 0.05	1.67 ± 0.05 ns	0.91 ± 0.02	2.4 ± 0.2 μs	0.085 ± 0.003

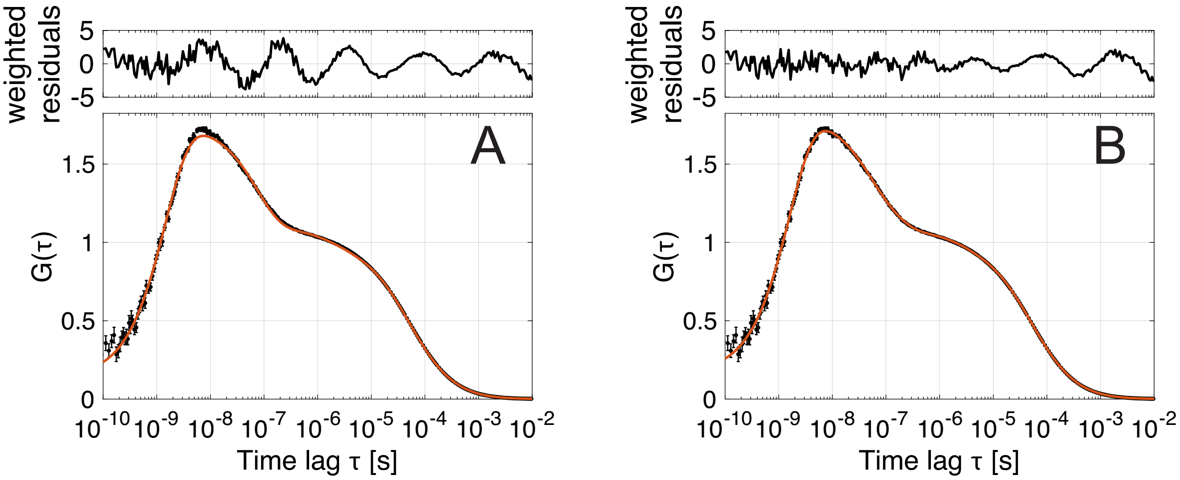

FIG. A. PET-FCS dynamics. Fit of the experimental correlation function using a model accounting for one kinetic component (A) and two kinetic components (B). The weighted residuals show that one kinetic component is not sufficient to describe the observed dynamics. The $\chi^2_{red.}$ goodness-of-fit measure changes from 2.80 to 1.09 upon inclusion of a second kinetic component. The Bayesian information criterion (BIC) is significantly lowered from 765 to 340, justifying the inclusion of the second kinetic component.

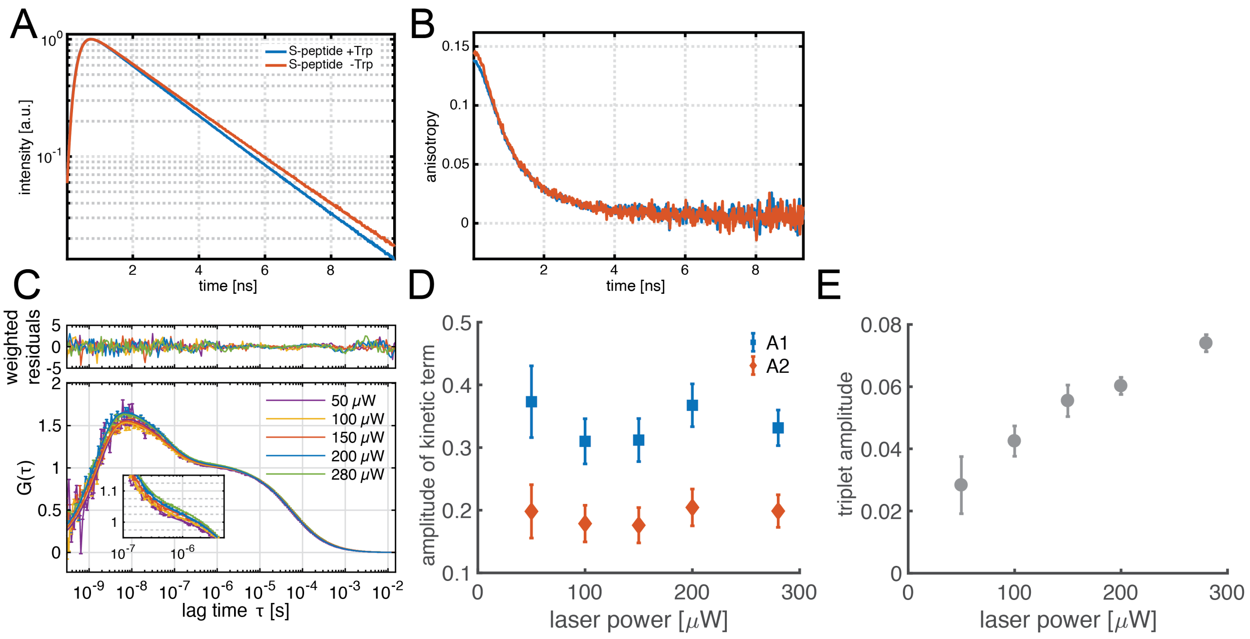

FIG. B. Control experiments. (A) Normalized intensity decays for S-peptide. The lifetime changes from 2.2 ns to 2.1 ns upon addition of the tryptophan residue. (The lifetime of free Atto655 dye is 1.8 ns) (B) The time-resolved fluorescence anisotropy shows no significant change. (C-E) Testing for power dependence of observed bunching terms reveals that only the triplet term at ∼3 μs depends on laser power, while the kinetic terms are not affected. (A1) Amplitude for ∼37 ns component. (A2) Amplitude for ∼120 ns component.
